# Comparison of the efficacy of nickel-titanium rotary systems with or without the retreatment instruments in the removal of gutta-percha in the apical third

**DOI:** 10.1186/1472-6831-14-102

**Published:** 2014-08-15

**Authors:** Flávio Rodrigues Ferreira Alves, Thiago Oliveira Ribeiro, Jaime Omar Moreno, Hélio Pereira Lopes

**Affiliations:** 1 Department of Endodontics, Faculty of Dentistry, Estácio de Sá University, Av. Alfredo Baltazar da Silveira, 580/cobertura, Recreio dos Bandeirantes, Rio de Janeiro, RJ 22790-710, Brazil; 2 Department of Endodontics, Santo Tomas University, Bucaramanga, Colombia

**Keywords:** Gutta-percha removal, Mtwo, ProTaper Universal, Root canal retreatment, Rotary NiTi instruments

## Abstract

**Background:**

The purpose of this study was to compare the efficacy of nickel-titanium rotary systems with or without the retreatment instruments in the removal of gutta-percha from the apical third.

**Methods:**

The systems compared were as follows: ProTaper Universal (PT), ProTaper Universal Retreatment (PTr), Mtwo (M2) and Mtwo Retreatment (M2r). Sixty extracted mandibular incisors were treated with a crown-down technique and filled with gutta-percha and sealer. The apical diameter was standardized in 0.30 mm, 1 mm from the apex. The teeth were distributed into 4 experimental groups: PT, PTr, M2 and M2r. In PTr and M2r groups, filling materials were removed by PTr/M2r followed by root canals preparation up to a PT F4/M2 40; in groups PT/M2, the filling materials were removed and the root canals were prepared by PT up to a PT F4/M2 up to a M2 40. The roots were split and photomicrographing. The percentage of clean area in the apical 5 mm was calculated using software. Data were analyzed with the Kruskal-Wallis test.

**Results:**

Remaining material was found in all hemisections and there was no statistically significant difference between the groups (p = 0.09). Considering the surface of the canal walls of all teeth, the mean of the percentage of clean area was 54%.

**Conclusions:**

Considering the applied methodology, remaining filling material was found in all hemisections, regardless of the retreatment technique and PT or M2 were as effective as PTr/PT or M2r/M2.

## Background

The main objective of endodontic retreatment is the same as any other form of treatment, to restore or prevent the health of the periapical tissues. Therefore, it is necessary to remove the filling material from root canals, to clean, to shape and to re-fill them. Rotary instrumentation systems have been applied in retreatment, not only for the reinstrumentation of root canals, but also for removal of filling material [1].

Some established instrumentation systems, such as ProTaper (PT) and Mtwo (M2), have recently introduced specific instruments for removing filling materials during retreatment. ProTaper Universal Retreatment System (PTr) has three instruments, D1-30/0.09, D2-25/0.08, and D3-20/0.07 and Mtwo Retreatment (M2r) has two instruments, R1-15/0.05 and R2-25/0.05. Few studies have compared the effectiveness of these retreatment systems. Bramante et al. [2] compared the removal of gutta-percha and zinc oxide and eugenol-based sealer provided by PTr, M2r and Hedström files by measuring of remaining material areas observed on a stereomicroscope (×12.5). Mtwo Retreatment files were less effective, leaving a significantly higher amount of filling material compared to the other instruments. PTr and hand files did not differ in the cleaning ability. Somma et al. [3] comparing the cleaning provided by the same instruments, founded that manual removal of different filling materials was more effective than rotary systems. Using a logistic regression model, the authors showed that the apical third of the root canal had the greatest impact on the score values. Marfisi et al. [4] found no significant differences in the area of remaining material, between PTr and M2r in roots filled with gutta-percha or resilon.

The removal of filling material simultaneously with the instrumentation has also been used for gutta-percha removal, using only the rotary instrumentation systems (and not the retreatment instruments) [5-7]. Tasdemir et al. [7] compared ProTaper, Mtwo, Hedström files and R-Endo (an exclusive system for retreatment) in removing gutta-percha. The teeth used in the study were rendered transparent and the area of remaining filling material was measured using computer software. PT was significantly more effective than M2 in cleaning. The other comparisons between the techniques were not statistically significant.

It is not yet established in literature if specific instruments for filling removal are essential for retreatment with NiTi rotary systems. Therefore, this *in vitro* study compared ProTaper and Mtwo efficacy, preceded or not by the retreatment instruments, in the removal of filling material from the apical third of root canals.

## Methods

Sixty human extracted mandibular incisors with completely developed apices were provided by Tooth Bank of Estácio de Sá University. The reasons for extraction were not related to this study, and the ethics committee of the Estácio de Sá University approved the research protocol (process number 0133.0.308.000-10). Bucco-lingual and mesio-distal radiographs were taken to confirm the existence of a single straight canal. The coronal access cavity was prepared using high-speed diamond burs under water spray.

### Initial preparation

The root canals were prepared with a crown-down instrumentation approach using the principles of the alternated rotary motions technique [8], through the following steps: 1. Canal negotiation and establishment of patency with #10-#20 stainless steel K-files (Maillefer, Ballaigues, VD, Switzerland); 2. Gates-Glidden burs (Maillefer, Ballaigues, VD, Switzerland) in the coronal two-thirds; 3. Working length (WL) determination at 1 mm from the apical foramen introducing a K-file #10 into the canal until it was visible at the foramen; 4. Hand stainless steel K-files preparation in the apical third up to a #30 mm in WL; 5. Step back with stainless steel K-files increasing the size of the instruments and backing up the length (in steps of 1 mm; 3–5 step-backs were sufficient to complete the preparation). During the preparation, abundant and frequent irrigation with 2.5% NaOCl (usually 1–2 mL after each file size) were performed. After irrigation, apical patency was verified by introducing a #10-#20 K-file in the canal until its tip was visualized at the apical foramen. Upon completion of chemomechanical preparation, smear layer was removed with a total of 5 ml of 17% EDTA (Biodinâmica, Ibiporã, PR, Brazil) for 3 min (renewing after each minute) and then the root canal was irrigated with 2.5% NaOCl again.

The teeth were dried with absorbent paper point and subsequently filled with laterally compacted gutta-percha, using a standard master cone size 30 (Dentsply, Petrópolis, RJ, Brazil), which was introduced 1 mm shorter of the apex, and fill canal sealer (TECHNEW, Rio de Janeiro, Brazil), a zinc oxide and eugenol-based sealer. The quality of the filling was checked through bucco-lingual and mesio-distal radiographs. The teeth were then stored in 100% humidity at 37°C for 14 days to allow for the sealer to set.

The teeth were transversely sectioned with a double-sided diamond disc at 15 mm from the apex. The purpose of this step was to establish a standard length.

The roots were randomly divided into 4 groups of 15 each as follows; group PTr, filling material was removed with PTr (Dentsply Maillefer, Ballaigues, VD, Switzerland) followed by preparation with the PT system (Dentsply Maillefer, Ballaigues, VD, Switzerland), up to F4-40/0.06-0.05-0.04-0.03; group PT, filling removal and reinstrumentation with PT system up to F4; group M2r, filling removal with M2r (VDW, Munich, BY, Germany) followed by preparation with M2 system (VDW, Munich, BY, Germany) up to F4-40/0.04; and group M2, filling removal and reinstrumentation with M2 system, up to file F4-40/0.04.

### Filling removal and reinstrumentation

The filling material was removed coronally to the extent of 2 mm deep by using a cylindrical diamond bur number 50 (KG Sorensen, Cotia, SP, Brazil). These cavities were filled with one drop of eucalyptol solvent (Biodinâmica, Ibiporã, PR, Brazil) using an insulin syringe, which was left in place for 3 min before starting filling removal.

The torque for all rotary instruments was 1.0 N.cm. The speed for retreatment instruments was 700 rpm and 300 rpm for the PT and M2. The movement applied to all rotary instruments was the enlargement with continuous rotation. After filling removal (PTr and M2r groups), the root canals were irrigated with 2 mL of 2.5% NaOCl to remove the debris formed. During the reinstrumentation (all groups), a pattern of irrigation with 2 mL of 2.5% NaOCl at each change of files was established. A hypodermic needle with 0.55 mm of diameter was used to allow penetration to the middle third of the roots. WL adopted for both filling removal and reinstrumentation was 1 mm short of the apex. The instruments were replaced by new ones every 5 roots.

All techniques were performed by a single operator. The work with one instrument was considered completed when file reached the working length, and there was no filling material covering the instrument. The smear layer was not removed after this step. The details for the instrumentation systems, used alone or combined are as follows.

### ProTaper universal retreatment

D1-30/0.09 file was used for removal of filling material from the root canals in the coronal third, penetrating 5 mm in apical direction. D2-25/0.08 file was used until to middle third, penetrating 10 mm. Finally, D3-20/0.07 file was used in the apical third, penetrating until the WL was reached. Manufacturer’s recommendations were followed: progressive advancement in apical direction with in and out motion combined with brushing, small decreases of 1 to 2 mm, frequent removal of the file to inspect it and removal of the propellers’ debris before continuing.

### ProTaper universal

The instruments S1-17/0.02-0.11 and S2-20/0.04-0.08-0.05 were used up to achieve the WL. After irrigation, F1-20/0.07-0.04 file was used to achieve the WL and the irrigation was performed again, followed by F2-25/0.08-0.04-0.03, F3-30/0.09-0.06-0.04-0.03 and F4-40/.06-.05-.04-.03 files, using the same criteria. The above recommendations were also followed.

### Mtwo retreatment

Mtwo size 15/0.05 was used until the WL, followed by 25/0.05 file until the WL, both with lateral pressure. Manufacturer’s recommendations were followed: frequent removal of the file to inspect it and removal of the propellers’ debris before continuing.

### Mtwo

Mtwo 10 file was introduced to achieve the WL and irrigation. Mtwo 15/0.05, 20/0.06, 25/0.06, 30/0.05, 35/0.04 and 40/0.04 were used in WL. The root canals were irrigated at each change of file. The same movement was used to ProTaper system.

### Evaluation

When the reinstrumentation was finished, the roots were maintained 7 days, at 37°C, for complete evaporation of the irrigant. For the later evaluation of exactly apical 5 mm (see below), the teeth were sectioned in 8 mm from the apex, approximately, perpendicular to the long axis, with a double-sided diamond disc, without refrigeration. The coronal portion of roots was discarded. After that, two longitudinal sections, centered on the proximal surfaces of the roots, were performed. A chisel was inserted into the grooves and the roots were split.

Photomicrographs were obtained from each apical hemisection (Leica DFC 290 camera HD; Leica Microsystems, Heerbrugg, St. Gallen, Switzerland) under a stereomicroscope at ×16 magnification (Leica LED3000 NVI; Leica Microsystems, Heerbrugg, St. Gallen, Switzerland). The images were captured and analyzed using Leica Application Suite 3.6.0 software. It was selected the tool *Region of Interest* to restrict the analysis to the apical 5 mm. Then, on the *Acquire* tab, it was defined 4 set points focus on *Steps* option. Finally, the *Create Multifocus After Align Stack* and the *Acquire Images Before Combining* were set to obtain a final combined image. The images were saved as TIFF format. The remaining filling material and the total canal area (mm^2^) were measured in the final combined images in both apical hemisections, only in the last apical 5 mm. A qualified examiner, blinded to the techniques used in the experiment, performed the measurements.

The clean area was calculated by subtracting the area containing the remaining filling material from the total canal area. The measurements of the clean area and the total canal area, for each hemisection, were added. With these data, the percentage of the clean area, considering the total canal area, was calculated. The measurements obtained for each NiTi rotary system were analyzed with the Kruskal-Wallis test. Statistical analyze was performed with SPSS version 17.0 (SPSS Inc, Chicago, IL, United States of America) with a confidence level set at 5%.

## Results

After filling removal and reinstrumentation, remaining filling material was found in all hemisections, regardless of the retreatment technique. The mean of the percentage of clean area was 54% considering the sixty instrumented teeth. The percentage of clean area was only higher than 50% in 34 teeth. The mean of the remaining filling area was 3.38 mm^2^ and the mean of the total canal area was 7.17 mm^2^. The root with less remaining filling material showed 0.40 mm^2^ (9% of the total area), while the highest amount found was 8.68 mm^2^ (79% of the total area). Considering the total canal areas of all sixty teeth, the root canals did not vary significantly between the groups (p =0.17).

The techniques did not differ significantly (p = 0.09) in percentage of clean area. The mean of the percentage of clean area, median, minimum, maximum and standard deviation in each group are shown in Table 1.

**Table 1:** Percentage of clean area: median, minimum, maximum and standard deviation in each group

**Group**	**Roots**	**Mean**	**Median**	**Minimum**	**Maximum**	**SD**
PTr	15	63.20	69.65	25.29	90.97	17.75
PT	15	49.95	50.34	20.26	67.44	15.42
M2r	15	50.02	49.28	25.14	84.33	16.10
M2	15	54.28	56.90	26.35	88.30	19.20

## Discussion

The maximum removal of gutta-percha and sealer followed by adequate reinstrumentation of root canals are important to the success of endodontic retreatment. Thereby, the clinician has better access to remnants of necrotic tissue and microorganisms that are causing the persistence of periapical inflammation [9]. The present study, like many others [2,6,7,9-11], also verified that the removal of filling in all root canals were not complete. Furthermore, the percentage of clean area was much lower than expected, only 54%.

The difference between the mean clean area percentages found in M2r and PTr groups were similar to those reported by Bramante et al. [2] in the evaluation of the apical third, and no statistically significant difference was found. These authors only compared retreatment instruments. In the present study, the difference between these groups was 13.18% (PTr = 63.20% and M2r = 50.02%) while in the aforementioned study was 14.90% (PTr = 47.30% and M2r = 32.40%). Certainly, the largest percentage of clean area found is justified by the fact that these groups were reinstrumented with both corresponding instrumentation systems up to 0.4 mm of diameter. Marfisi et al. [4] also found no significant difference when comparing both retreatment instruments, independently of the analyzed third. The difference between the percentage means to the clean area was very small, less than 1% (PTr = 76.30% and M2r = 75.67%) in the CBCT analysis.

In the present study, the removal of filling material simultaneously to the instrumentation (PT and M2 groups) did not differ among themselves, but the group M2 left a smaller amount of remaining material compared to the PT group (3.26 mm^2^ and 4.14 mm^2^, respectively) considering the absolute values. This result contradicts the findings of Tasdemir et al. [7] who found that the PT system (final instrument, F3-30/0.09-0.05) showed significantly lower mean of remaining material compared to the M2 system (final instrument, F4-30/0.05).

Besides the D0 of the last instrument, the use of solvent and the irrigation were also standardized. The application of solvent during root canal retreatment is controversy. It is known that solvents have been used to facilitate the process, but these should be used with care given their cytotoxic potential and the possibility of forming a residual film of softened gutta-percha on the root canal walls [12,13]. Taking it into account, in the present study, the solvent was restricted to the coronal portion of the gutta-percha, just to facilitate the initial penetration on the gutta-percha and the volume was minimal. Differences in the volume of solvent or irrigant would certainly influence the cleaning during retreatment.

Following other studies [2,5,9-14] the present study used the percentage of clean area because this analysis takes into account the total area of the canals, a variable that certainly influences the quality of cleaning. Some studies, however, have measured only the amount of remaining material [3,6,7,15]. The method used for evaluation was the longitudinal cleavage and quantitative analysis with a stereomicroscope. This method allow a direct visualization of remaining filling material with magnification and it is a well established method in the literature [1,9,16]. Other common methods for this type of investigation are: radiographs [6,17], rendered transparent teeth [7,15], CBCT [4] and computed microtomography [10]. Among these, only mCT seems superior to the method employed by us. With radiographs and cleared teeth, the overlapping areas of remaining material are a common problem.

The question why the tested instruments sequences and many others of previous studies are unable to remove all remains of filling material needs to be addressed. The main reason for this occurrence lies in the fact that most endodontic instruments do not fit the root canal walls. Complementary procedures, as the use of Self Adjusting File or filing with Hedström files, have been employed to try solving this problem [18,19]. However, studies need to be conducted to develop a sequence or an instrument to optimizing the filling removal.

The design of this study was crucial to answer the question if retreatment instruments (Group PTr and M2r) are really necessary for the removal of filling material. It was evident that the instruments of two tested systems have similar performance to conventional NiTi rotary instrumentation systems (Group PT and M2), since there was no significant difference between tested groups. Furthermore, taking into account that in the present study, the number of instruments ranged 6 to 9 depending of the group, the number of instruments in a retreatment technique may not have great influence on gutta-percha removal. This is in agreement with a recent study that did not find statistical difference comparing two single file techniques with PTr in gutta-percha removal [13].

## Conclusions

Considering the applied methodology, remaining filling material was found in all hemisections, regardless of the retreatment technique and PT or M2 were as effective as PTr/PT or M2r/M2.

## Competing interests

The authors declare that they have no competing interests.

## Authors’ contributions

FRFA contributed supervising the study, writing and reviewing the manuscript. HPL designed the study. TOR was the specialist who performed the retreatment techniques and everything related to teeth operation. JOM performed the stereomicroscope analysis. All authors read and approved the final manuscript.

## Authors’ information

FRFA is PhD and Professor from Department of Endodontics, Faculty of Dentistry, Estácio de Sá University, Rio de Janeiro, RJ, Brazil. TOR is specialist in Endodontics and Postgraduate Student from Department of Endodontics, Faculty of Dentistry, Estácio de Sá University, Rio de Janeiro, RJ. JOM is Professor from Department of Endodontics, Santo Tomas University, Bucaramanga, Colombia. HPL is a Dr. and Professor from Department of Endodontics, Faculty of Dentistry, Estácio de Sá University, Rio de Janeiro, RJ, Brazil.

## Pre-publication history

The pre-publication history for this paper can be accessed here:

http://www.biomedcentral.com/1472-6831/14/102/prepub
